# Sublinear drag regime at mesoscopic scales in viscoelastic materials

**DOI:** 10.1371/journal.pone.0299296

**Published:** 2024-03-07

**Authors:** A. E. O. Ferreira, J. L. B. de Araújo, W. P. Ferreira, J. S. de Sousa, C. L. N. Oliveira

**Affiliations:** 1 Departamento de Física, Universidade Federal do Ceará, Fortaleza, Ceará, Brazil; 2 Laboratório de Ciência de Dados e Inteligência Artificial, Universidade de Fortaleza, Fortaleza, Ceará, Brazil; Beijing University of Technology, CHINA

## Abstract

Stressed soft materials commonly present viscoelastic signatures in the form of power-law or exponential decay. Although exponential responses are the most common, power-law time dependencies arise peculiarly in complex soft materials such as living cells. Understanding the microscale mechanisms that drive rheologic behaviors at the macroscale shall be transformative in fields such as material design and bioengineering. Using an elastic network model of macromolecules immersed in a viscous fluid, we numerically reproduce those characteristic viscoelastic relaxations and show how the microscopic interactions determine the rheologic response. The macromolecules, represented by particles in the network, interact with neighbors through a spring constant *k* and with fluid through a non-linear drag regime. The dissipative force is given by *γv*^*α*^, where *v* is the particle’s velocity, and *γ* and *α* are mesoscopic parameters. Physically, the sublinear regime of the drag forces is related to micro-deformations of the macromolecules, while *α* ≥ 1 represents rigid cases. We obtain exponential or power-law relaxations or a transitional behavior between them by changing *k*, *γ*, and *α*. We find that exponential decays are indeed the most common behavior. However, power laws may arise when forces between the macromolecules and the fluid are sublinear. Our findings show that in materials not too soft not too elastic, the rheological responses are entirely controlled by *α* in the sublinear regime. More specifically, power-law responses arise for 0.3 ⪅ *α* ⪅ 0.45, while exponential responses for small and large values of *α*, namely, 0.0 ⪅ *α* ⪅ 0.2 and 0.55 ⪅ *α* ⪅ 1.0.

## Introduction

Purely elastic and purely viscous behaviors are limited cases of constitutive equations of materials [1]. Actual substances may deform and flow, but one of these attributes usually dominates the other, depending on the applied conditions. This solid-liquid duality has teased researchers since at least the 19^th^ century. Back then, pioneers such as James Maxwell and Ludwig Boltzmann proposed analytical models based on series and parallel associations of springs and dashpots to explain the peculiar characteristics observed in silk, glass fibers, and steel wires [2, 3]. The effective response of such early models invariably presents exponential relaxation decays, regardless of how springs and dashpots are connected. However, these simple approaches are only suited for some viscoelastic materials nowadays. In modern society, soft matter has become more common and accessible to everyone because we manufacture synthetic materials and probe samples that we were incapable of working with before, like living cells.

The emergence of such complex materials has triggered new theoretical models and the improvement of proper experimental techniques to explain and control their viscoelastic properties [4–8]. Nanoindentation methods, such as Atomic Force Microscopy, have become essential to characterize viscoelastic features at micro and nanometer scales by probing materials with nano-sized indenters [9]. The way the contact force changes on time reveals rheologic signatures of the material. The characterization of viscoelastic materials attempts to determine the relaxation function *R*(*t*) that possesses both qualitative and quantitative information.

Exponential and power-law relaxation functions are the two major types of experimentally probed responses. Polyacrylamide gels [10, 11] and aqueous solutions of cationic surfactants [12], for instance, present exponential-like response, *R*(*t*) ∝ *e*^−*t*/*τ*^, with a relaxation time *τ* for the material to achieve a new equilibrium configuration. On the other hand, living cells [13, 14], microgel dispersions [15], soft glassy materials [16], collagen gels [17] and hydrogels [18] present a time-dependent power-law-like behavior, *R*(*t*) ∝ *t*^−*β*^. Both types of relaxation dynamics were observed in functionalized colloids on attractive substrates [19, 20]. As observed in elastic materials [21, 22], macroscopic physical parameters are intrinsically connected to their microscopic interactions, structures, and timescales [23–25].

Power laws and exponentials arise in many physical phenomena having a deep origin in their dynamic processes. For instance, in non-additive entropy systems, many physical variables are described by power-law distributions instead of the traditional exponential functions in the counterpart entropy [26–28]. Exponential and power-law canonical distributions emerge naturally regarding whether the heat capacity of the heat bath is constant or diverges [29]. Moreover, power laws are associated with emergence phenomena where their exponents display scaling behaviors near criticality [30, 31]. Systems with the same critical exponents belong to the same universality class, and a small set of universality classes describes almost all material phase transitions.

One of the challenges in material science is linking the physical mechanisms at microscopic scales to macroscopic functional behavior. This approach is especially relevant for soft matter because properties on the molecular scale are linked to conformational and compositional fluctuations on the nanometer and micrometer scale and, in addition, span many orders of magnitude in length [32, 33]. Soft matter holds rich structures and various interactions at the mesoscale, where thermal energy per unit volume is negligible, in contrast with the high energy density stored in atomic bonds of crystalline structures [34]. While exponential materials can be modeled by an association of springs and dashpots, such as the so-called standard linear solid model, power-law materials are usually obtained by fractional rheology [35, 36] or glassy rheology models [37, 38]. However, these models cannot explain the connection between macroscopic responses and their elastic and viscous components. Multiscale approaches may help reveal the material’s constitutive relations regarding its minor scale interaction and composition.

Here, we design a model of viscoelastic materials composed of an immersed elastic network of macromolecules to study how mesoscopic interactions influence macroscopic rheological behavior in soft materials [39]. We assume non-linear hydrodynamic drag forces act between the macromolecule and the fluid, where the contribution of elastic and viscous interactions are controlled at the mesoscopic level. By changing the physical parameters of elastic and drag forces, we obtain materials with exponential or power-law relaxations or an intermediary behavior for responses that are not clearly characterized. Our results show that exponential behavior is, in fact, the most common regime of deformation, being described by the standard linear solid model [39]. Power-law responses are exceptional outcomes for a particular range of sublinear drag forces.

## Numerical model for viscoelastic materials

**Fig 1:**
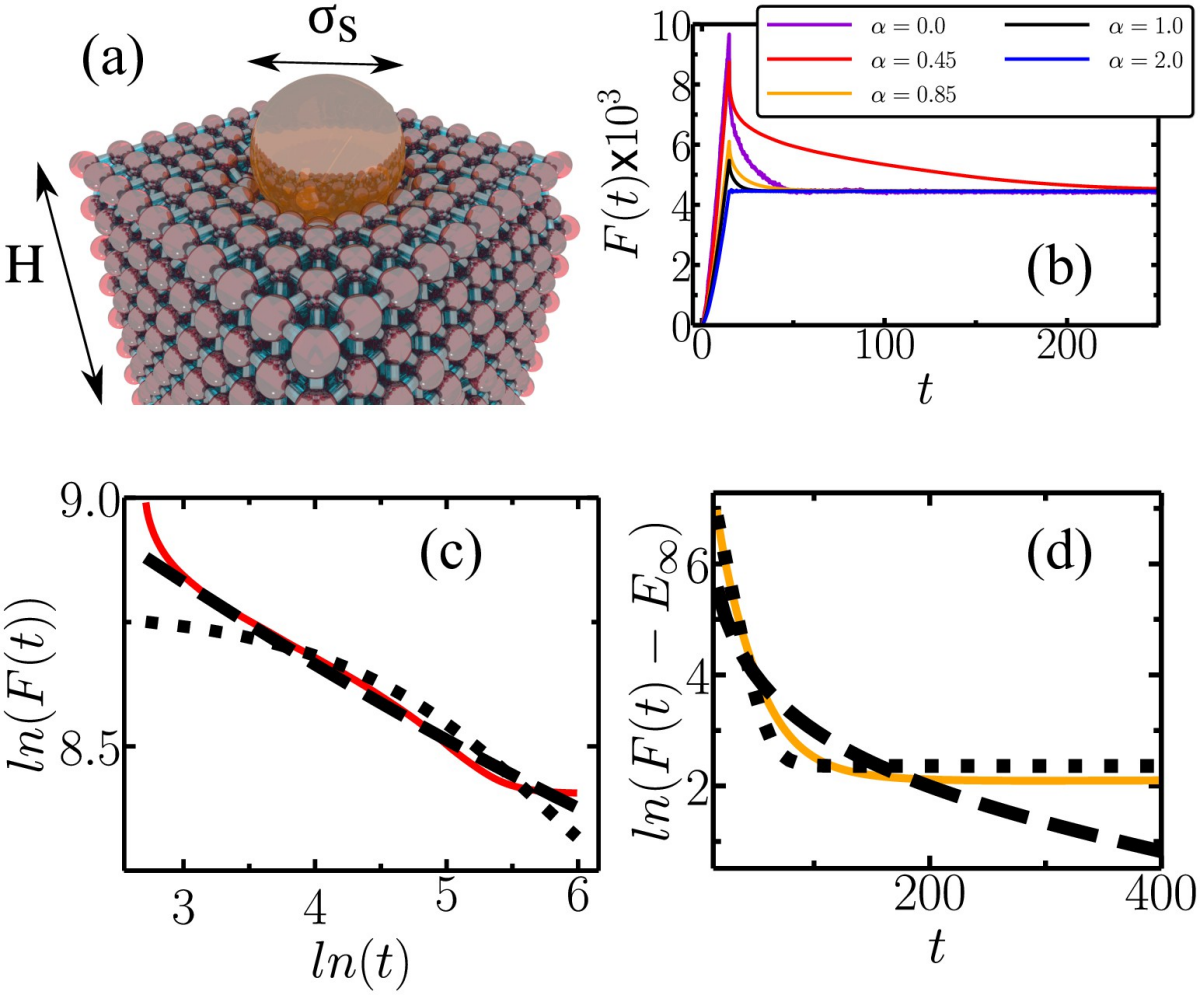
(a) Viscoelastic model made by a face-centered cubic (FCC) lattice of macromolecules immersed in a viscous fluid (not shown in the image). Each spherical particle of diameter *σ* represents a collection of molecules interacting with an effective spring constant *k* with its twelve closest neighbors. A rigid spherical punch of diameter *σ*_*s*_ indents the network from top to bottom. *H* is the number of layers in the vertical direction. (b) Contact force curves for *γ* = 80 and *k* = 800 and several values of *α* exhibiting different viscoelastic relaxations. The cases for *α* = 0.45 and *α* = 0.85 are shown in panels (c) and (d), respectively, where the dashed and the dotted lines represent fit with the exponential and power-law model of Eqs 5 and 6.

Our model consists of *N* spherical particles of diameter *σ* and mass *m* arranged in a face-centered cubic (FCC) lattice with dimensions *x*×*y*×*z* given by *σH*sin(*π*/3)×*σH*sin(*π*/3)×*σH*, where *H* is the number of layers in *z* direction, as shown in Fig 1(a). Every particle interacts with its twelve nearest neighbors through an elastic potential with an effective spring constant *k*. The spring network is immersed in a viscous fluid, where drag forces act on moving particles. In this coarse-grain approach, the particles may represent macromolecules found in suspended polymer chains, colloidal aggregations, and other load-bearing structures of soft matter.

We perform computational indentation assays to probe the network’s effective viscoelastic properties. Firstly, a rigid spherical indenter presses down the network at a constant rate. This *loading stage* is done during a time *τ*_*l*_ until a maximum indentation depth *δ*_*max*_ is achieved. After that the indenter stays still while the network rearranges towards a minimum energy configuration, which is called *dwell* stage. Particles in the bottom layer are not allowed to move along the *z*-axis (the direction of the stress applied) but are free to slide horizontally. We limit the maximum indentation to less than 10% of the network height to avoid finite-size effects [40], meaning that *δ*_*max*_ ≈ *σ*. The Hookean interaction between macromolecules (represented by particles in the network) is not generally true. However, considering small deformation, this assumption should be valid for any well-shaped potential (Lennard-jones potential, for instance) but not for charged particles where forces are monotonic. Slight indentations also prevent particles from behaving in a jammed state [41–43]. Moreover, future works should investigate the role of irregular networks, rather than an FCC lattice, in their viscoelastic response.

The equation of motion of the *i*_*th*_ particle, at position ${\vec{r}}_{i}$, is given by the following equation [44]

$$\begin{array}{c} m\frac{d^{2}{\vec{r}}_{i}}{dt^{2}}=-\nabla U_{i}-\gamma v_{i}^{\alpha }{\hat{v}}_{i}, \end{array}$$ (1)

where *U*_*i*_ is the interaction potential of particle *i* due to other particles and the indenter, given by

$$\begin{array}{c} U_{i}=\frac{k}{2}\sum_{j}{\left(r_{ij}-\ell \right)}^{2}+\epsilon {\left[\frac{\sigma }{r_{is}-\left(\sigma_{s}-\sigma \right)/2}\right]}^{\xi }. \end{array}$$ (2)

The summation in the first part runs over the neighbors, where $r_{ij}=\left|{\vec{r}}_{i}-{\vec{r}}_{j}\right|$ and *ℓ* are, respectively, the distance and the equilibrium distance between the centers of particles *i* and *j*. The last term in Eq (2) represents a hard-core potential applied only to those particles in contact with the indenter, where *ϵ* is an energy parameter, and $r_{is}=\left|{\vec{r}}_{i}-{\vec{r}}_{s}\right|$ is the distance between the center of the particle and the indenter. The exponent *ξ* must be large enough to keep the stiffness of the indenter and to guarantee the hard-core potential acts only on the contact between particles. Here we use *ξ* = 400.

The last term of Eq (1) represents a generalized drag force acting oppositely to the particle velocity, ${\vec{v}}_{i}=v_{i}{\hat{v}}_{i}$, with magnitude given by $\gamma v_{i}^{\alpha }$, where *γ* and *α* are related to the particle geometry and the fluid properties in which the particles are immersed. Dissipation vanishes for *γ* = 0, leading to purely elastic networks where our model reproduces the well-known Hertz behavior for mechanical contacts [39]. However, when local friction becomes relevant, *γ* > 0, the model may produce distinguished behaviors of viscoelastic materials. Notice that *α* = 1 and *α* = 2 represent typical values for drag forces acting on rigid structures. The linear regime, known as Stoke’s law, arises for small Reynolds numbers when viscous forces dominate over inertial forces, where *γ* is proportional to the medium’s viscosity and the particle’s diameter. On the other hand, the quadratic drag is dominant for large Reynolds numbers. In this case, *γ* is proportional to the medium’s density and the cross-sectional area of the particle.

The physical origins of the sublinear regime (*α* < 1), however, are entirely different and are related to the deformability/adaptability of bodies subjected to drag forces [45]. Many living beings present sublinear drag behaviors, where deformability is a survival strategy to protect their fragile structures under hydrodynamic conditions. Characterizing the drag exponents in deformable systems is recurrent in botany, aerodynamics, and hydrodynamics [46–49]. Typical drag exponents for algae are as small as 0.34 [50]. On the other hand, tulip and willow oak trees are more rigid and present exponents close to unity with *α* = 0.82 and *α* = 0.94 [47], respectively. The limiting case of *α* = 0 corresponds to constant frictional forces, regardless of the particle velocity.

## Results and discussions

The numerical solution of Eq (1) for *N* = 2748, *ℓ* = 1.1*σ* and *H* = 15*σ* is performed through molecular dynamics simulations [51, 52] with periodic boundary conditions applied to the horizontal plane, and time integration is done with the velocity Verlet algorithm with time step *dt*. The force is computed as the sum of all collisions on the indenter at each time step. On continuum elastic samples, the Hertz model for mechanical contact states that the contact force is a power-law of the indentation, *f* ≈ *δ*^λ^, where the exponent λ depends on the geometry of the indenter. Usual values are 1, 3/2, and 2 for flat, spherical, and conical indenters, respectively. In our network model, however, the ratio between *σ*_*s*_ and *σ* changes how the network perceives the deformation. For indenters as big as the particles (*σ*_*s*_ ≈ *σ*), the Hertz exponent approaches 2, as deformations done by a conical indenter on continuum samples. For indenters much greater than the particles (*σ*_*s*_ ≫ *σ*), the exponent tends to 1, as deformations done by a flat indenter. The Hertz exponent equals 3/2 (as expected for a spherical indenter) only for intermediate sizes of the indenters, specifically, for *σ*_*s*_ = 11*σ*, which is the value used in this work. The relation between λ and *σ*_*s*_ for different numbers of particles is presented in [39]. See [53] for the parameters used. Each set of mesoscopic parameters, *k*, *γ*, and *α*, represents a specific material and defines the macroscopic viscoelastic responses. To investigate how microscopic properties lead to the rheological behavior of the entire network, we perform simulations varying the spring constant between 100 and 1000, the drag constant between 10 and 100, and the drag exponent between 0.0 and 1.0, totalizing 2100 different networks.

Fig 1(b) shows typical force curves for *γ* = 80 and *k* = 800 and different values of the drag exponent. *F*(*t*) is the computational analogue of an indentation assay used to characterize macroscopic responses of actual materials. In the loading stage, the indenter slowly deforms the network until the contact force reaches its maximum value, which is inversely proportional to *α*. In the dwell stage, *F*(*t*) relaxes until part of the mechanical energy is lost through friction. The timescale of such dissipation depends on *α* in a highly complex fashion. For *α* = 2, dissipation is fast enough so that the response behavior is qualitatively identical to a perfectly elastic network. For *α* < 1, drag forces slowly remove energy from the network leading to a viscoelastic decay.

In a continuum approach, the analytical time-dependent contact force of a viscoelastic sample indented by a spherical punch follows the convolution integral [39]

$$\begin{array}{c} F\left(t\right)=\int_{0}^{t}R\left(t-t^{\prime }\right)\frac{d\delta^{\frac{3}{2}}\left(t^{\prime }\right)}{dt^{\prime }}dt^{\prime }, \end{array}$$ (3)

where *δ*(*t*) is the indentation depth history. The contact force in Eq (3) is normalized by the constant $4\sqrt{\sigma_{s}}\delta_{max}^{\frac{3}{2}}/3\left(1-\nu^{2}\right)$, where *ν* = 0.5 is the Poisson coefficient. Typical relaxation behaviors for viscoelastic materials are given by

$$\begin{array}{ccc} R_{P}\left(t\right) & = & E_{\infty }+\Delta E\;\;t^{-\beta },\\ R_{E}\left(t\right) & = & E_{\infty }+\Delta E\;\;e^{-\frac{t}{\tau }}, \end{array}$$ (4)

where *R*_*P*_(*t*) is the relaxation model for power-law materials with an exponent *β* and *R*_*E*_(*t*) for exponential materials with relaxation time *τ*. In both models, *E*_∞_ is the elastic modulus at considerable times when the material is completely relaxed, and Δ*E* is the difference between the maximum value, at *t* = *τ*_*l*_, and *E*_∞_. We assume that materials become perfectly elastic after some time, although this long-time elasticity plateau is only sometimes observed in actual materials. This assumption is necessary to avoid high-pressure effects in our numerical model. For instance, if the external force is large enough to ultimately compress the small particles until a condensate network is formed, any additional pressure would induce the deformation of those particles. However, micro-deformations are allowed only due to the motion of the pseudo-atoms, which is represented by the sublinear drag regime. Solving Eq (3) with Eq (4) in the dwell stage leads, respectively, to

$$\begin{array}{ccc} F_{P}\left(t\right) & = & E_{\infty }+a\;t^{-\beta }, \end{array}$$ (5)

$$\begin{array}{ccc} F_{E}\left(t\right) & = & E_{\infty }+b\;e^{-\frac{\left(t-\tau_{l}\right)}{\tau }}-c\;e^{-\frac{t}{\tau }}, \end{array}$$ (6)

where the constant $a\approx \frac{\Delta E}{\Gamma (1-\beta )}$ is obtained by expanding the incomplete beta function, Γ(1 − *β*) is the gamma function, and $b=\frac{3\tau }{2\tau_{l}}\Delta E$ and $c=\frac{3\sqrt{\pi }}{4}{\left(\frac{\tau }{\tau_{l}}\right)}^{\frac{3}{2}}\Delta E\;\text{erfi}\left(\sqrt{\frac{\tau_{l}}{\tau }}\right)$, where erfi($\sqrt{\frac{\tau_{l}}{\tau }}$) is the imaginary error function.

Fitting the dwell part of the force curve allows us to map the macroscopic mechanical properties (*E*_∞_, Δ*E*, *τ*, *β*) with the mesoscopic parameters (*k*, *γ* and *α*) as done previously for *α* = 1 [39]. Here we focus on finding the qualitative rheological behavior rather than describing relations among parameters. Fig 1(c) and 1(d) show the same force curves as in (b) for *α* = 0.45 and *α* = 0.85, respectively, where the curves are fitted both with $F_{P}\left(t\right)$ and $F_{E}\left(t\right)$. Clearly, the *α* = 0.45 case is better fitted with the power-law model, while *α* = 0.85 with an exponential.

**Fig 2:**
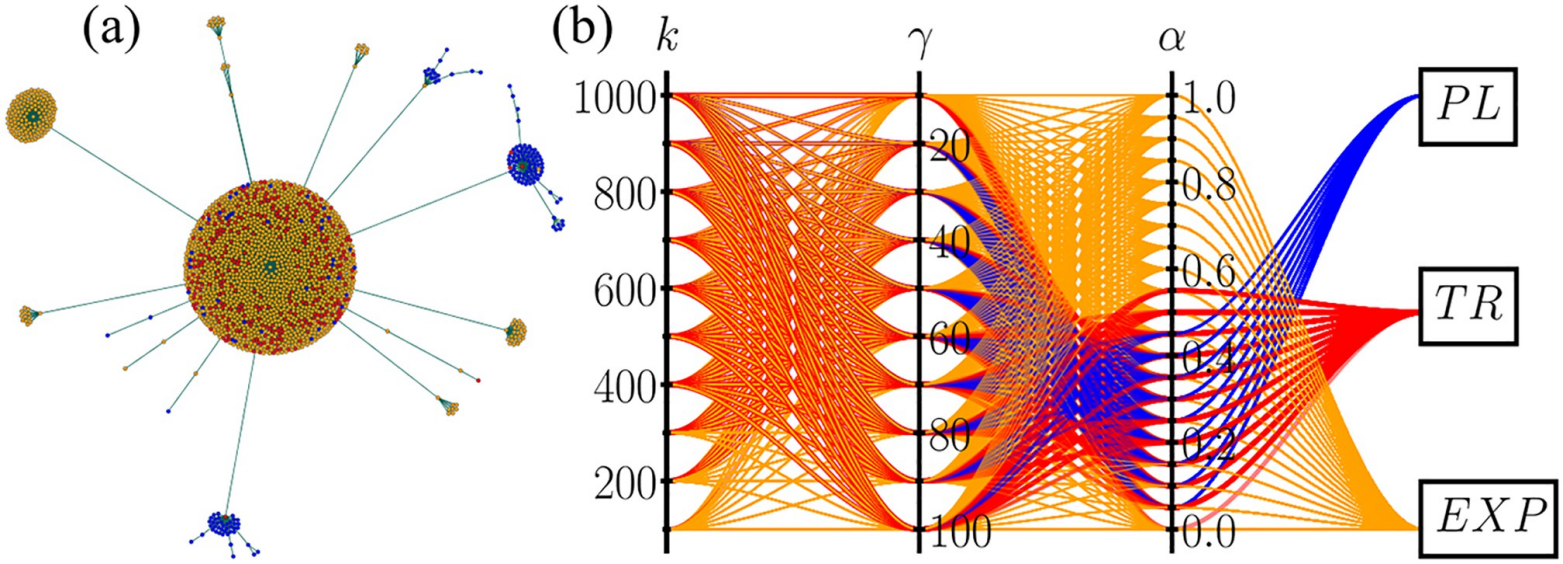
(a) Graph visualization of the clustering process using *K-means* method. Each dot represents a computational experiment for a given combination of *k*, *γ*, and *α*, and the colors represent different relaxation outcomes, namely, power-law (PL) in blue, exponential (EXP) in yellow, and transitional behavior (TR) in red. (b) A parallel plot shows how different values of the mesoscopic network parameters lead to different types of viscoelastic relaxation. Each line crosses a combination of *k*, *γ*, *α*, and the corresponding rheological behavior.

The mean-square error determines the goodness-of-fit parameter between the obtained numerical force curve *F*(*t*) and the analytical force models given in Eqs 5 and 6,

$$\begin{array}{c} \chi_{M}=\frac{1}{N_{p}}\sum_{i=1}^{N_{p}}{\left[F\left(t_{i}\right)-F_{M}\left(t_{i}\right)\right]}^{2}, \end{array}$$ (7)

where *N*_*p*_ is the number of points in the force curves and *M* stands for exponential (*E*) or power-law (*P*) model type. This statistical index, calculated for each combination of *k*, *γ*, *α*, is used in a *K-means* method as an unsupervised clustering strategy [54, 55] to classify the computational force curves as either an exponential or a power-law behavior, or even a transitional regime that cannot be clearly classified. In this machine learning process, the classification of the material takes into account not only the values of *χ*_*E*_ and *χ*_*P*_ but also the trends and distributions in the phase space of *χ*_*E*_ and *χ*_*P*_. Fig 2(a) shows the graph visualization of this clustering process. The exponential behavior is indeed the most common one, as shown in the big cluster in Fig 2(a), but small groups of power-law materials are also observed. The parallel coordinates plot in Fig 2(b) summarizes the rheological outcomes. Each line passes through every combination of *k*, *γ*, and *α* ending up in one of the three boxes representing the response behavior of the network.

**Fig 3:**
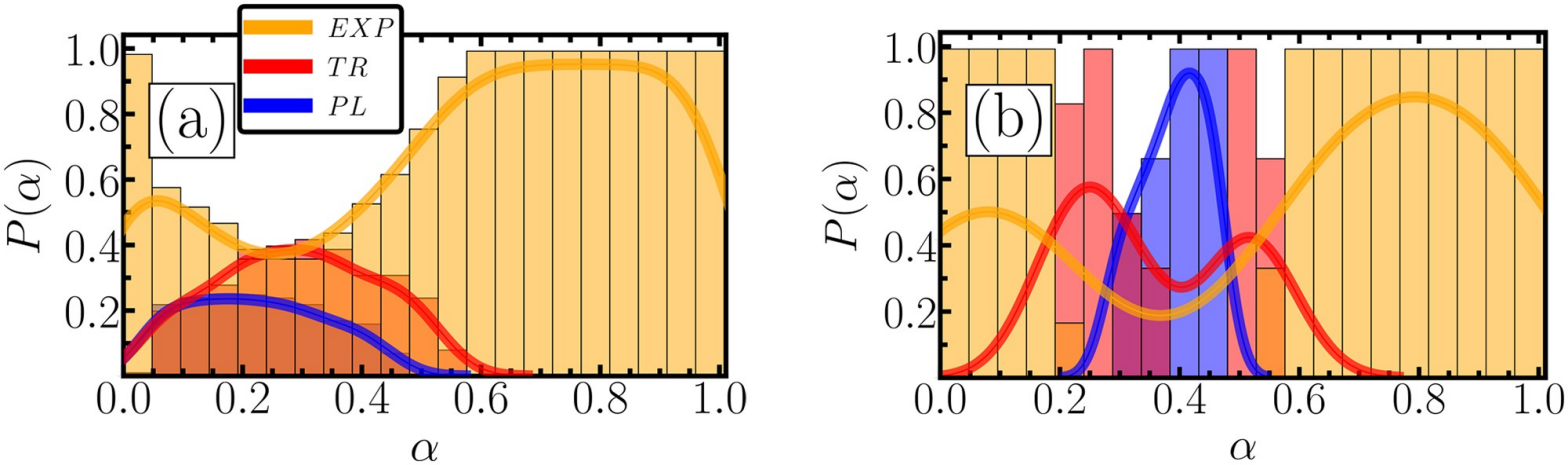
The probability that a given *α* leads to a power-law (blue bars), an exponential (yellow bars), or a transitional response (red bars). The solid lines show the probability density computed by the KDE (kernel density estimation) algorithm. Panel (a) shows results for the whole range of *k* and *γ*, while panel (b) considers only networks with *k* ≥ 800 and *γ* ≥ 80.

To understand why small clusters of power-law materials form in Fig 2(a), we must investigate the impact of considering sublinear drag regimes. Fig 3 shows the normalized probability *P*(*α*) of finding PL, EXP, or TR behavior for a given *α*. In panel (a), *P*(*α*) is computed for all values of *k* and *γ* considered here. The three distributions strongly overlap, making classification a challenging exercise. In panel (b), on the other hand, we remove small values of *k* and *γ*. For networks not too soft, *k* ≥ 800, and not too elastic, *γ* ≥ 80, those distributions split for different regions of *α*, and the drag exponent becomes the central controller to characterize the macroscopic behavior. Exponential behaviors are found mainly for *α* ⪅ 0.2 and *α* ⪆ 0.55, while the relaxation is a power law for *α* between 0.3 and 0.45. For intersecting values of *α*, the superposition of the probabilities of PL and EXP leaves doubt in classifying the material either as an exponential or a power law. The effect of Stokes drag forces (for *α* = 1) between macromolecules and the solvent they are immersed in play a role in the viscoelasticity properties of colloidal and polymeric materials [56–58]. However, to our knowledge, our work is the first to explain the underlying mechanics that control the viscoelastic response in soft matter in terms of sublinear drags. Recent works have used modified drag forces in mesoscale interactions of soft matter but again with different intentions [59, 60].

**Fig 4:**
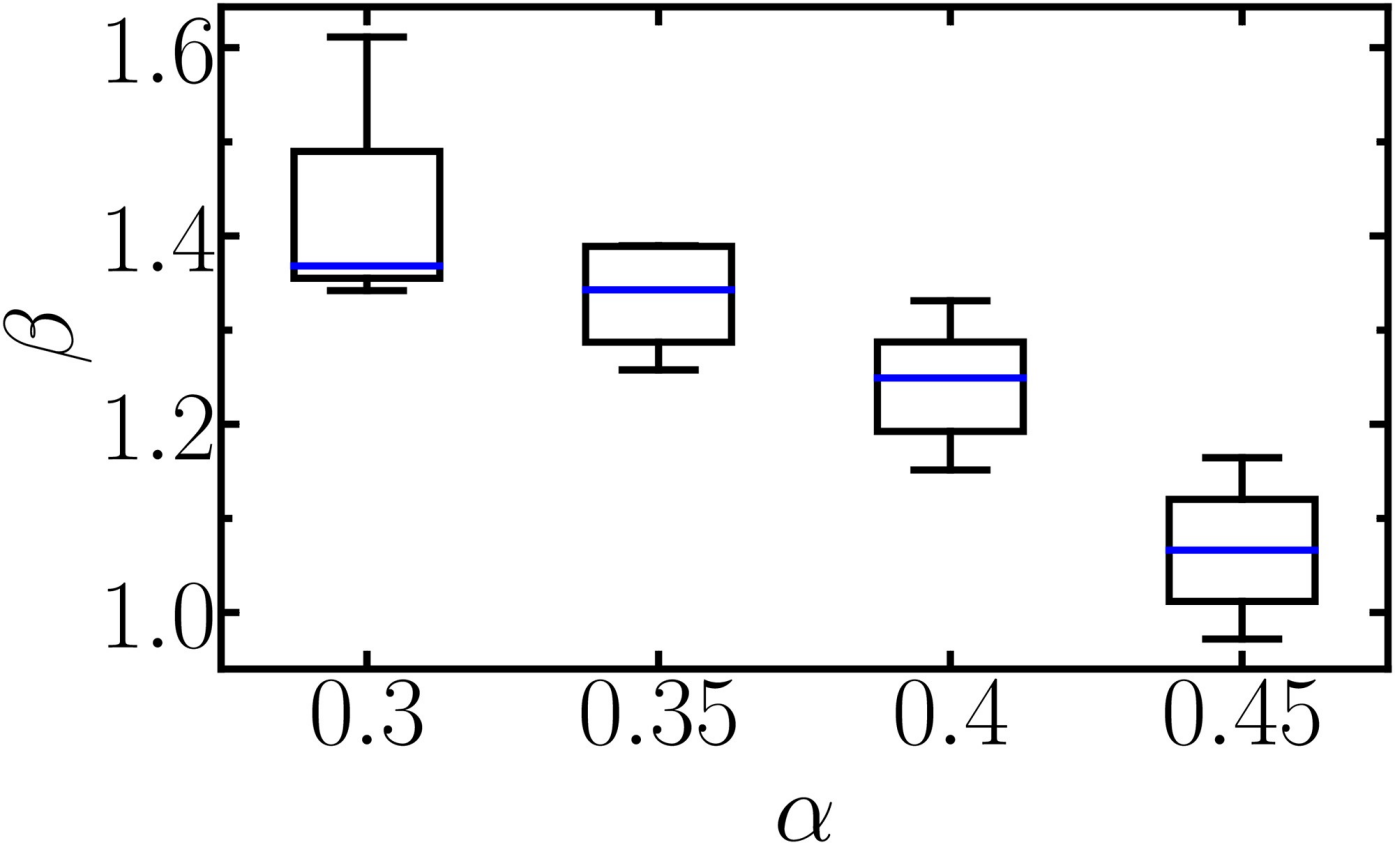
For those networks classified as a power-law material in Fig 2, we show the relationship between the relaxation exponent *β* and the mesoscopic drag exponent *α*.

For those cases where the network presents a power-law behavior, we show in Fig 4 the relationship between the macroscopic relaxation exponent *β* and the drag exponent. For each value of *α*, there is a small dispersion distribution of *β* whose mean value lies between 1.05 and 1.35. Actual materials exhibiting power-law relaxation usually present smaller exponents and exhibit structural disorder and metastability [36–38, 61]. Computational investigations of disordered two-dimensional networks obtained relaxation exponents between 0.5 and 0.75, depending on the network arrangement [25]. Our simulations exhibit macroscopic relaxation exponents above 1.0, mainly because our viscoelastic solid model is structurally ordered and stable, restricting the viscoelastic responses to faster relaxation regimes.

## Conclusion

In conclusion, we show why viscoelastic materials present power-law or exponential relaxation responses. Using molecular dynamics simulations, we perform numerical indentations onto a macromolecular network immersed in a fluid and reproduce typical viscoelastic signatures as those found experimentally. The macromolecules interact with each other through an elastic potential and with the fluid through a non-linear drag regime given by *γv*^*α*^, where *γ* and *α* are viscous parameters, and *v* is the particle’s velocity. Our results show that these three mesoscopic parameters control the viscoelasticity response of the entire network. We classify the macroscopic viscoelasticity for each set of interacting parameters using an unsupervised clustering algorithm according to the deformed network’s relaxation type: exponential relaxation, power-law relaxation, or a transitional behavior between them. In the *α* = 1 case, the drag force is proportional to the particle velocity, while for *α* = 0, the drag force is constant. These two limits of *α* always lead to exponential responses, regardless of the other two mesoscopic parameters. Although exponential behaviors are the most frequent, power-law viscoelasticity arises for specific values of those interacting parameters. If we disregard small *k* and small *γ*, i.e., for networks that are neither too soft nor too elastic, power-law responses are solely determined by the viscous exponent for 0.3 ⪅ *α* ⪅ 0.45. When *α* changes from 0.3 to 0.45, the power-law relaxation exponent *β* decreases from 1.38 to 1.07. Part of the energy that would dissipate for *α* = 1 seems to be used for the macromolecules (such as colloids and polymers) to undergo deformation due to the conformational changes in the sublinear case. More investigation should still be performed using techniques such as Fluid-Structure Interaction, where the fluid and elastic dynamic equations are coupled. However, the consumed energy for macromolecule deformations seems to be the physical explanation for the power-law viscoelasticity.
